# Bibliographical Notices

**Published:** 1844-08-10

**Authors:** 


Report on the Sanitory condition of the Laboring Population of Great Britain.

*A Supplementary Report on the results of a special inquiry into the practice of Interment in towns. Made at the request of her Majesty's principal Secretary of State for the home department.* By Edwin Chadwick, Barrister at Law. Presented to both Houses of Parliament, by command of her Majesty. 8vo. pp. 279. London, 1843.

This is one of the most deeply interesting publications that we have read for a long time. It abounds in facts well authenticated, not only in relation to the propriety of interments in towns, but likewise as to many of the evils arising from an overcrowded population. It exhibits in this last respect, a condition of society in the great metropolis of England, of which the inhabitants of this bountiful land have no conception.

According to Mr. Chadwick, "in a large proportion of cases in the metropolis, (London,) and in some of the manufacturing districts, one room serves for one family of the labouring classes; it is their bed-room, their kitchen, their work-house, their dining room; and, when they do not follow any out-door occupation, it is frequently their work-room and their shop. In this one room they are born, and live, and sleep, and die amidst the other inmates." According to an inquiry made by Mr. Weld, secretary of the statistical society, at the instance and expense of Lord Sandon, as to the condition of the working classes resident in the inner ward of St. George's, Hanover Square, and in the immediate vicinity of some of the most opulent residences in the metropolis, it appeared that 1465 families of the labouring classes had for their residence 2175 rooms, and 2510 beds. "Out of 5945 persons, 839 were found to be ill, and yet the season was not unhealthy. One family in eleven had a third room (and that not unoccupied) in which to place a corpse. This, however, appears to be a favourable specimen. From an examination made by a committee of the statistical society into the condition of the poorer classes in the borough of Mary le bone, it appeared that the distribution of rooms amongst the portion of population examined, shewed that not more than one family in a hundred had a third room.

| | Families. | Single persons. |
|---|---|---|
| No. occupying part of a room, | 159 | 196 |
| " one room, | 382 | 56 |
| " two rooms, | 61 | 2 |
| " three rooms, | 5 | 7 |
| " four rooms, | 1 | 0 |

"Mr. Leonard, Surgeon and Medical Officer of the parish of St. Martins-in-the-Fields, gives the following instances of the circumstances in which the poorest class of inhabitants die, which may be adduced as exemplifications of the dreadful state of circumstances in which the survivors are placed for want of adequate accommodation for the remains immediately after death, and previous to interment:—

'There are some houses in my district that have from 45 to 60 persons of all ages under one roof, and in the event of death, the body occupies the only bed till they raise money to pay for a coffin, which is often several days.' 'Some live in kitchens under ground, under the Adelphi arches, and in the dampest and worst ventilated situations.' 'Of course, says our author, the tenants are never free from fevers and diarrhœa, and the mortality is great. The last class live, for the most part, in lodging rooms, where shelter is obtained, with a bed of straw, for from 2d. to 4d. per night, and when this is not obtained, the arches under the Adelphi afford a shelter. In the lodging rooms, I have seen the beds placed so close together as not to allow room to pass between them, and occupied by both sexes indiscriminately. I have known six people sleep in a room about nine feet square, with only one small window, about fifteen inches by twelve inches; and there are some sleeping rooms in this district in which you can scarcely see your hand at noonday!'" p. 32.

The great point of inquiry in this report relates to the innocuousness or otherwise of putrid emanations upon human health. In opposition to M. Parent Duchâtelet and others, our author maintains the doctrine that all such emanations, whether from human remains or the remains of inferior animals, are decidedly prejudicial. In support of this view, he has collected many important facts, indisputably established as such, while he has examined and confuted with great cleverness most of the statements adduced in support of the opposite side of the question. The most common forms of disease originating from this cause, are fevers and intestinal derangements, especially the latter. It appears that not only does the human constitution suffer from such exposure, but likewise beasts and birds. Cattle confined in slaughter houses lose their appetites and become sick, and birds so exposed sicken and die. We have been particularly struck with the following statement: A bird-fancier who lived near Clare-market (London,) in a situation particularly "exposed to the combined effluvia from a slaughter house and a tripe factory, found he could not rear his birds in this place." Birds fresh from the country would die in a week. "He had previously lived for a time in the same neighbourhood in a room over a crowded burial ground in Portugal street; at times in the morning he had seen a mist rise from the ground, and the smell was offensive. That place was equally fatal to his birds." On removing to another situation not exposed to such emanations, he was again able to raise birds.

We have heretofore believed that putrid emanations arising from animal decomposition, unless concentrated in a high degree, were rather offensive than deleterious; or at least that their noxiousness was much over-rated; but we must confess that the evidence and arguments adduced by Mr. Chadwick have greatly shaken our confidence in this opinion. He has indeed proved to our entire conviction that interments ought not to be permitted in populous towns. He adduces the strongest evidence, not only that the water in wells in the vicinity of grave yards becomes contaminated, but that in many instances the effluvia or gaseous products of decomposition, pass upward through the superincumbent earth to an amount sufficiently great to be detected by the most ordinary means. This takes place more readily in dry and sandy than in clay soils.

"It has been considered that all danger from interments in towns would be obviated, if no burials were allowed except at a depth of five feet. But bodies buried much deeper are found to decay; and so certain as a body has wasted or disappeared, is the fact that a deleterious gas has escaped. In the towns where the grave yards and streets are paved, the morbific matter must be diffused more widely through the sub-soil, and escape with the drainage. * * * * Dr. Reid detected the escape of deleterious miasma from graves of more than 20 feet deep." p. 28.

---

Human Health; *or the influence of atmosphere and locality; change of air and climate; seasons; food; clothing; bathing and mineral springs; exercise; sleep; corporeal and intellectual pursuits, &c. &c., on healthy man; constituting Elements of Hygiène.* By Robley Dunglison, M. D., Professor of the Institutes of Medicine in Jefferson Medical College, &c. *A new edition, with many modifications and additions.* 8vo. pp. 464. Philadelphia, Lea & Blanchard. 1844.

This is a *second edition* of the author's work on hygiène, recast, as well as revised, and consequently materially modified as to its original character. Of late years the whole subject of hygiène, in its public and private relations, has been the subject of much attention on the part of philanthropists and enlightened legislators. "The results of the researches of able investigators have modified materially many of the views which had been generally entertained in regard to the salubrity or insalubrity of different callings, and have led to a greater degree of attention on the part of philanthropists to the domestic condition of the poorer classes, to which, rather than their industrial relations, it would seem that many of the evils must be ascribed."

The author has considered the various subjects mentioned in the title, it appears to us, fully and fairly, and stated, without undue bias, the facts and opinions most entitled to our confidence on the several points. He is decidedly favourable to rural cemeteries, and in that respect coincides with Mr. Chadwick in deprecating "intra-mural interments," although he can scarcely be said to go all lengths with him in his views of the pernicious influence of putrid emanations. He rejects altogether the opinion that the emanations from either animal or vegetable matters, in a state of putrefaction, occasion malaria,—meaning thereby the cause of intermittent fever,—although he admits the injurious effects of continual exposure to such influences upon the general health of most individuals.

We cannot enter upon an analysis of this work, or examine in detail any of the chapters which it contains, without transcending our limits. We can, however, very cheerfully recommend it to the attention of readers generally, lay as well as professional, for the great amount of useful information which it contains upon subjects of deep concernment to all classes. The style of the author is so well known, that we need hardly speak of the present work in that respect, further than to remark that, although less technical, and less concisely written than most of his productions, it is on these accounts more readable for the general inquirer, and no less instructive for those better acquainted with such subjects.

